# Prostheses for the will

**DOI:** 10.3389/fnsys.2014.00079

**Published:** 2014-05-08

**Authors:** Walter Glannon

**Affiliations:** Department of Philosophy/Arts, University of CalgaryCalgary, AB, Canada

**Keywords:** brain-computer interface (BCI), deep brain stimulation (DBS), hippocampal prosthesis, neural circuits, neurological disorders, psychiatric disorders, will

## Introduction: the neurobiology of the will

The will is a complex set of cognitive (including affective and motivational) and motor capacities that enable us to initiate and complete action plans. Neurological and psychiatric disorders impair these capacities because of dysfunction in neural circuits mediating them. Parkinson's disease (PD), major depressive disorder (MDD), obsessive-compulsive disorder (OCD), and other conditions can be understood as disorders of the will involving overactive, underactive or inactive critical nodes of these circuits (Lozano and Lipsman, 2013). Neural prostheses such as brain-computer interfaces (BCIs), hippocampal prostheses (HPs), and deep-brain stimulation (DBS) can bypass, replace, or modulate damaged or dysfunctional circuits and thereby restore or enhance the capacities necessary to translate intentions into actions. In this respect, they can be described as prostheses for the will.

Philosophers claim that for the will to be free, actions must not be generated by causal routes that bypass and undermine agent's control of the mental states that issue in them (Mele, 1995). Presumably, this would include manipulation of the brain by an artificial implanted device. Yet neural prostheses that bypass, replace, or modulate dysfunctional circuits do not undermine but instead restore this control when it has been lost and can enhance it when it is impaired by brain injury or neurodegeneration. They restore control of thought and behavior by restoring the relevant motor and mental functions. I will describe the different respects in which the three neural prostheses in question can achieve this goal.

## Brain-computer interfaces: restoration of motor function

BCIs utilize operant conditioning and goal-directed thinking in restoring some degree of motor function. As motor prostheses, they can enable some individuals with paralysis caused by traumatic brain injury, neurodegenerative disease, or limb loss to move a cursor on a computer screen or a robotic arm by detecting signals in the motor cortex associated with intending to perform these actions. These systems may involve macroelectrodes placed on the scalp and connected to an EEG, electrodes implanted subdurally or epidurally, or a microelectrode array implanted in the motor cortex. What causes the cursor or robotic arm to move is the mental act of forming and executing an intention by the person manipulating the interface. BCIs may also enable those with disorders of consciousness or locked-in syndrome who retain a high level of cognitive functioning to reliably communicate their wishes about continuing or discontinuing life-sustaining treatment when they cannot communicate behaviorally. Difficulties with learning how to manipulate BCIs, sustaining attention to the task at hand, and the semantic capacity necessary to communicate for those with cognitive impairment are some of the challenges presented by this technology (Birbaumer et al., 2014). Whether a person using such a system can translate his or her thoughts into actions may depend on the extent of brain injury, which cortical circuits are intact, and how effective the practitioner is in training the patient to operate the interface. The extent to which the patient can do this successfully, and thus the extent to which he or she can exercise the motor component of the will, can be a matter of degree.

## Hippocampal prostheses: restoration of memory encoding

HPs to restore the cognitive function of memory have been used as prototypes in animal models but have not yet been used in humans. While they are at the developmental stage and may be ready for implantation in the human brain in the next 5 years, they remain a hypothetical intervention. Electrical stimulation of the fornix, which projects to a circuit consisting of the hippocampus and entorhinal cortex, improved semantic, working, and procedural memory in at least one person in a Phase I trial for early-stage Alzheimer's disease (Laxton et al., 2010). For those with this or other dementias whose hippocampal degeneration is too advanced to respond to neurostimulation, or for those with anterograde amnesia from traumatic injury to the hippocampal-entorhinal circuit, an HP replacing it might be able to restore the ability to encode new memories and learn and retain information (Berger et al., 2011; Hampson et al., 2013). It would do this by re-establishing inputs and outputs in this circuit. This structure is a component of the episodic memory system and one of the first structures to undergo cellular loss and tau pathology in Alzheimer's disease. Artificial reconstruction of neuron-to-neuron connections with a biomimetic microchip model replacing the hippocampal-entorhinal circuit could improve and sustain semantic, working, and procedural memory in those with degeneration in brain regions mediating memory and enable them to continue performing cognitive and physical functions. Memory provides a cognitive basis on which to imagine counterfactual and possible courses of action in the present and future (Hassabis et al., 2007; Schacter and Addis, 2007). It thus plays a critical role in deliberation and decision-making. A prosthesis that could resolve anterograde amnesia by re-establishing the ability to encode, consolidate, and retrieve episodic memory could restore planning and decisional capacity and restore one component of the will.

Theoretically, it would not matter whether memory functions were maintained by a natural or artificial system, provided that the prosthesis connected in the right way with the neural inputs and outputs necessary for these functions. To a certain extent, memory retrieval is an involuntary process. We have some degree of control of this process insofar as we can make some episodic memories consciously accessible and use the information in working memory for immediate cognitive demands and in prospective memory to plan ahead. But memories that flooded our brains with information serving no such purpose could be a burden and an impediment to free action. Device makers and practitioners implanting and activating an HP would have to ensure that circuits mediating the encoding, consolidation and retrieval of episodic memory were neither underactive nor overactive. They would also have to ensure that the device integrated with adjacent circuits in the medial temporal lobes and did not interfere with nondeclarative memory systems such as striatum- and cerebellum-mediated procedural memory and amygdala- and brainstem-mediated emotional context memory. In addition, the encoding function of an HP would have to be compatible with the meaning the agent assigns to past events and memories of them. This meaning influences how the agent imagines future situations and forms action plans. Prostheses would not assign meaning to newly formed memories but would encode them with equal value-neutral weight. This could impair goal-directed behavior if it interfered with the person's capacity to select some past events as more valuable than others in deliberating about courses of action. The encoding of new memories by the prosthesis would have to complement meaningful long-term memories that already have been encoded and consolidated and are available for retrieval. So the HP would not only have to integrate with other circuits in the person's brain but also with his or her history as an agent with a past and future.

## Deep brain stimulation: restoration of motor and cognitive functions

DBS has the widest range of applications among neural prostheses, restoring or enhancing motor as well as cognitive functions. It can be used as both as a probe and modulator of activity in dysfunctional neural circuits implicated in neurological and psychiatric disorders (Lozano and Lipsman, 2013). DBS has confirmed the pathophysiology of PD as degeneration of dopaminergic structures in the nigro-striatal pathway of the basal ganglia. Unilateral or bilateral stimulation of the subthalamic nucleus (STN) or globus pallidus interna (GPi) have restored circuit integrity and resulted in significant improvement for many patients with PD and other movement disorders such as primary dystonia and essential tremor in their ability to perform voluntary bodily movements. While the technique is still experimental and investigational for psychiatric disorders, DBS has confirmed that dysfunction in brainstem dopaminergic structures such as the ventral tegmental area (VTA) associated with motivation and reward is implicated in depression with symptoms of anhedonia and avolition. Stimulation of the nucleus accumbens, which receives projections from the VTA, has resulted in relief of these symptoms in some patients whose depression had been resistant to pharmacological treatment (Schlaepfer et al., 2008). By restoring their capacity to consider and engage in pleasurable activities, DBS can restore one component of their will. In addition, because of its projections to frontal-striatal pathways mediating cognitive, affective, and motor functions, stimulation of the STN can modulate dysfunction in these pathways and release individuals with OCD from paralyzing obsessions and compulsions (Mallet et al., 2008).

The neuromodulating effects of DBS can re-establish and sustain optimal levels of neural function, preventing extremes of deficit and surfeit and promoting flexible behavior and adaptability to the environment. Overstimulating targeted circuits, or inadvertently stimulating the wrong circuits, in an attempt to release mental and physical constraints caused by one type of neuropathology could cause a different type and have an equally disabling effect on the will. In PD, for example, electrical stimulation of the STN or GPi can resolve hypodopaminergic activity causing motor, cognitive, and emotional inhibition. But imprecise stimulation or overstimulation of these brain regions may induce hyperdopaminergic activity and produce behavioral disinhibition and impulsive or addictive behavior (Castrioto et al., 2014). The ethical implications of altering brain circuits with this technique in terms of benefit and harm are evident in reports of Parkinson's patients experiencing both symptom relief and neurological and psychological sequelae (Muller and Christen, 2011; Christen et al., 2012). Motor and emotional effects of stimulating the STN or GPi can be difficult to dissociate because the basal ganglia include these motor nodes as well as limbic nodes. There is considerable overlap between them, with afferent inputs and efferent outputs regulated by the same neurotransmitter. Similarly, in MDD overstimulation of an underactive nucleus accumbens associated with anhedonia and avolition can cause hyperdopaminergic effects in the reward circuitry and result in pathological behavior in the form of euphoria, hypomania, and mania (Synofzik et al., 2012). The cost-benefit ratio of DBS for depression and other psychiatric disorders is still unclear. The increasing use of this and other brain-invasive technologies raises ethical questions because not all outcomes are positive. A recent study of DBS of the reward system for MDD showed promising results within a few days of stimulation at lower intensities. As in earlier studies, however, surgical risks such as intracerebral hemorrhage and psychiatric complications such as suicidal ideation and hypomania in some subjects, as well as the high cost, were among the negative aspects of the technique (Schlaepfer et al., 2014). Some sequelae are not foreseeable, and it cannot be predicted which patients will experience them. This underscores the fact that this type of neuromodulation for psychiatric disorders remains experimental and investigational. Neural targets of DBS must be carefully selected and stimulation parameters adjusted in response to brain changes and neurological and psychiatric symptoms to maintain optimal levels of neural and mental function. Stimulation must sustain the neural and psychological mean between extremes.

Technological advances such as closed-loop feedback devices that can monitor and adjust to changes in the brain while circuits are stimulated can reduce the incidence of adverse effects. This would make them more tailored to individualized therapy in maximizing benefit and minimizing harm regarding the capacity to think and act. A recent study demonstrating positive outcomes from DBS at an early stage of PD suggests that it might have similar outcomes in treating psychiatric disorders if the effects on neural circuits are similar (Schuepbach et al., 2013). Electrical stimulation of dysfunctional circuits at an early stage of degeneration could strengthen synaptic connectivity, release trophic factors and possibly induce neurogenesis. This would be especially welcome in light of previous studies indicating that DBS for advanced neurodegenerative disorders can relieve symptoms but not alter the underlying pathology and disease progression. Even if DBS or other neural prostheses failed to arrest neurodegeneration, they might be able to delay or prevent further degeneration and enable patients affected by these disorders to retain some degree of control of their behavior by sustaining a certain level of cognitive and motor functions for longer periods.

## Conclusion

The ability of DBS to modulate underactive and overactive circuits in neurological and psychiatric disorders can restore or enhance the physical and mental capacities composing the will. Although their range of applications is more limited, BCIs and HPs could bypass or replace dysfunctional pathways in cortical and subcortical areas mediating motor control and dysfunctional pathways in the medial temporal lobes mediating episodic memory. A BCI may restore the will to some degree by enabling individuals paralyzed from brain or spinal cord injuries to translate their thoughts into certain actions and communicate their wishes about medical treatment. An HP replacing a damaged hippocampal-entorhinal circuit might enable individuals to learn and retain information necessary for present cognitive tasks and future planning. These considerations about the neurobiological basis of the will and the effects of altering it show that there is much in common between neuroscience and philosophy and that each discipline can inform and be informed by the other. In different respects and in varying degrees, the three prostheses I have discussed can re-establish and sustain the structural and functional integrity of the brain circuits necessary for freely willed actions.

### Conflict of interest statement

The author declares that the research was conducted in the absence of any commercial or financial relationships that could be construed as a potential conflict of interest.
